# Visual Loss As Primary Manifestation of Olfactory Groove Meningioma

**DOI:** 10.7759/cureus.37632

**Published:** 2023-04-16

**Authors:** Farhana Nabila Sulaiman, Mohamed iliyas Sultan Abdul Kader, Rajasudha Sawri Rajan, Wan Haslina Wan Abdul Halim

**Affiliations:** 1 Department of Ophthalmology, Faculty of Medicine, Universiti Kebangsaan Malaysia, Kuala Lumpur, MYS; 2 Department of Ophthalmology, Hospital Selayang, Batu Caves, MYS; 3 Department of Otorhinolaryngology-Head and Neck Surgery, Faculty of Medicine, Universiti Kebangsaan Malaysia, Kuala Lumpur, MYS; 4 Department of Otorhinolaryngology-Head and Neck Surgery, Hospital Melaka, Melaka, MYS

**Keywords:** intracranial tumours, optic disc swelling, meningoma, olfactory groove, visual loss, anosmia

## Abstract

Differential diagnosis of vision loss in a space-occupying lesion can be exhaustive. Olfactory groove meningioma (OGM) is a rare, benign, slow-growing tumour originating from the anterior cranial base. OGM is one of the differential diagnoses of intracranial tumours. We report a case of an OGM compressing the optic nerve and frontal lobe causing bilateral vision loss for six months. Multidisciplinary management by ophthalmologists, neurosurgeons, radiologists, and pathologists led to the diagnosis and tumour resection of OGM in the patient. Possible mechanisms of vision loss, imaging features, and treatment are discussed in this report.

## Introduction

Olfactory groove meningioma (OGM) comprises 5-18% of all intracranial meningiomas and is more common in women [1,2]. Most OGM are sporadic in nature. However, it may be associated with certain risk factors such as ionizing radiation exposure, hormonal factors, and obesity [3]. The gradual compression on the frontal lobes and a possible extension towards the sella by OGM can compress the optic nerve and optic chiasm resulting in visual loss [4]. We present a case of an OGM in an elderly male patient, compressing the optic nerve and frontal lobe causing bilateral vision loss for six months. Diagnosis and management in literature are discussed.

This article was previously presented as a meeting abstract at the 6th Asia-Pacific Glaucoma Congress 2022 in conjunction with the 12th Malaysian Society of Ophthalmology Annual Scientific Meeting (MSO-ASM) and the 36th Malaysia-Singapore Joint Ophthalmic Congress (MSJOC) on August 4-7, 2022.

## Case presentation

A 58-year-old male with no comorbidity presented to the eye clinic with a bilateral, gradual onset, generalised blurring of vision for six months which was progressively worsening for three months. It was associated with loss of smell. He had a significant weight loss of 10 kilograms within three months. This was the first episode of symptoms. There was no history of ocular trauma or family history of ocular diseases. He sought medical treatment after having recurrent falls at home due to his poor vision.

The patient had difficulty in the comprehension of instructions and commands. Ocular examination in the left eye revealed light perception vision and reduced colour discrimination with a positive relative afferent pupillary defect. The left optic disc was swollen with the blurring of the superonasal disc margin. The right optic disc was normal. Both eyes' anterior segment and intraocular pressures were normal with immature cataracts. There was no anisocoria noted.

Humphrey visual field (HVF) 24-2 test revealed bilateral complete visual field defect. Cranial nerve examination showed impaired cranial nerve I and II. On gait assessment, the patient had difficulty walking and required assistance likely due to poor vision. Other neurological and systemic examinations were unremarkable. Haematological and biochemical laboratory investigations such as full blood count, renal profile, liver function test and fasting blood glucose were normal.

**Figure 1:**
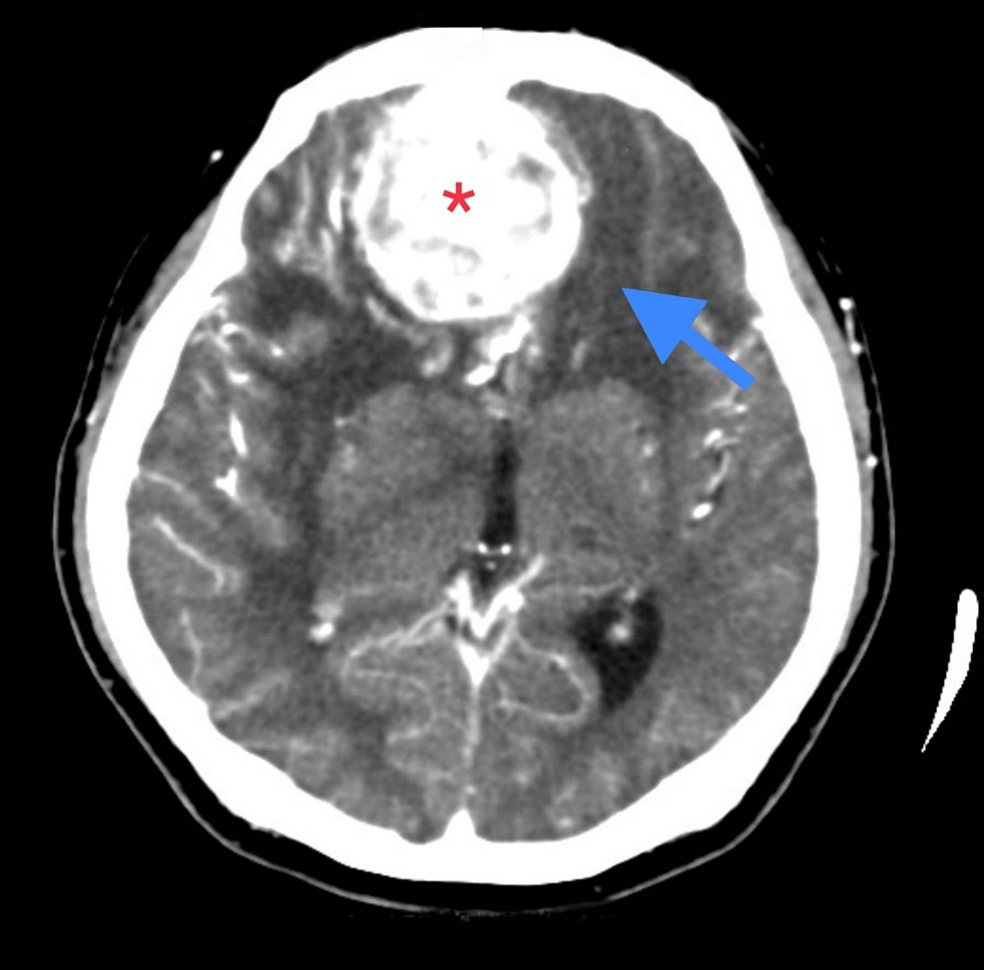
CECT Brain (axial view) showing bi-frontal extra-axial space-occupying lesion (red asterisk) measuring 4.8 x 5.0 x 4.8 cm with skull base erosion. There is a presence of perilesional oedema (blue arrow), left midline shift with ipsilateral ventricle compression. CECT: Contrast-enhanced computed tomography

**Figure 2:**
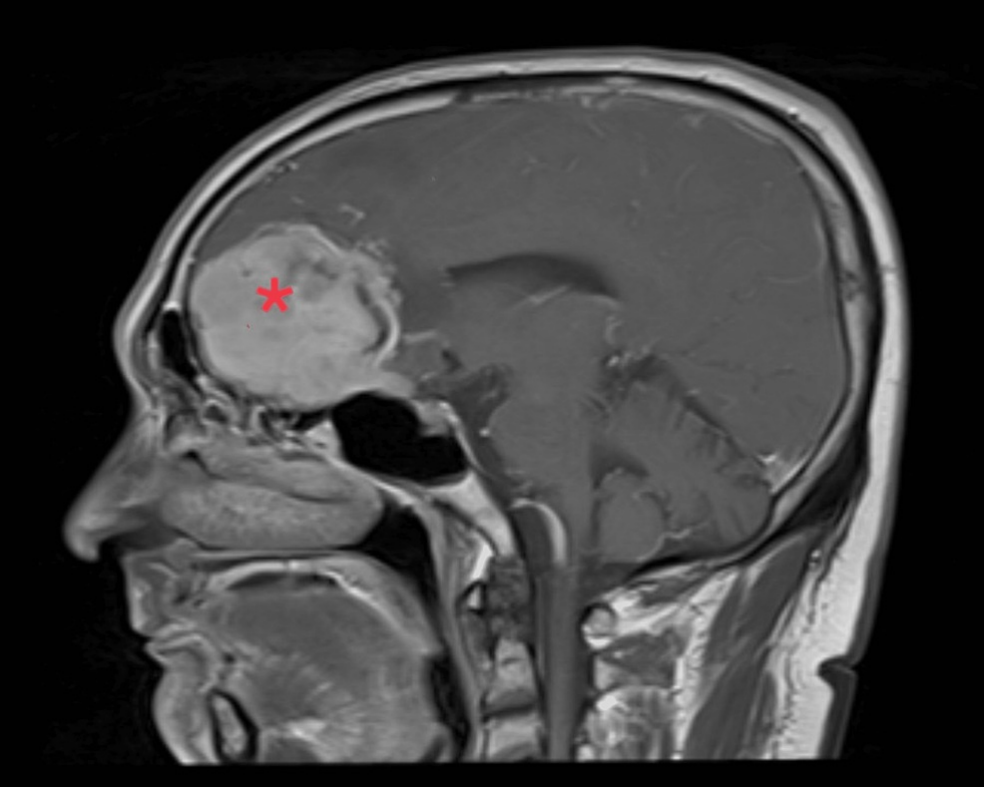
MRI (sagittal view) showing the anterior skull base meningioma (red asterisk) causing mass effect (right more than left), left midline shift, and contralateral early hydrocephalus.

Contrast-enhanced computed tomography (CECT) showed a bi-frontal extra-axial space-occupying lesion measuring 4.8 x 5.0 x 4.8 cm with skull base erosion (Figure 1). Magnetic resonance imaging (MRI), magnetic resonance angiography (MRA), and magnetic resonance venography (MRV) with intravenous gadolinium showed features of an anterior skull base meningioma causing mass effect (right more than left), left midline shift and contralateral early hydrocephalus (Figure 2). He was referred to the Neurosurgery team and underwent bi-frontal craniotomy and tumour de-bulking surgery. Intra-operatively, the tumour was moderately vascularized and rubbery in nature.

**Figure 3:**
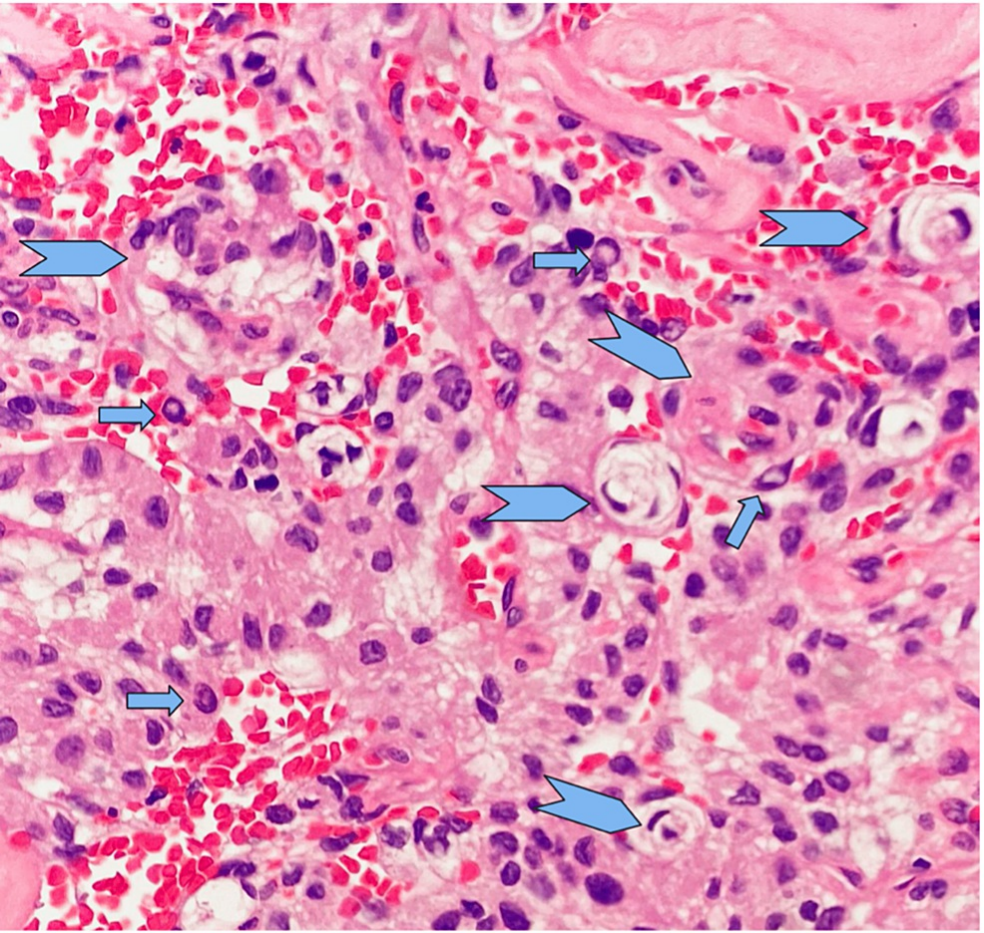
A generally uniform oval nucleus with a central clearing (arrow) and an indistinct cytoplasmic border. In areas, vague whorls of tumour cells are also present (arrow head).

Histopathology examination of the brain tissue confirmed meningioma, favouring angiomatous type (WHO Grade I). The histology section in Figure 3 shows tissues composed of numerous blood vessels intermingled with tumour cells displaying a generally uniform oval nucleus with a central clearing and an indistinct cytoplasmic border. The vascular channels are variable in size with a thickened hyalinised wall. In areas, vague whorls of tumour cells are also present. There is no significant pleomorphism noted. The mitotic figure is rarely seen.

**Figure 4:**
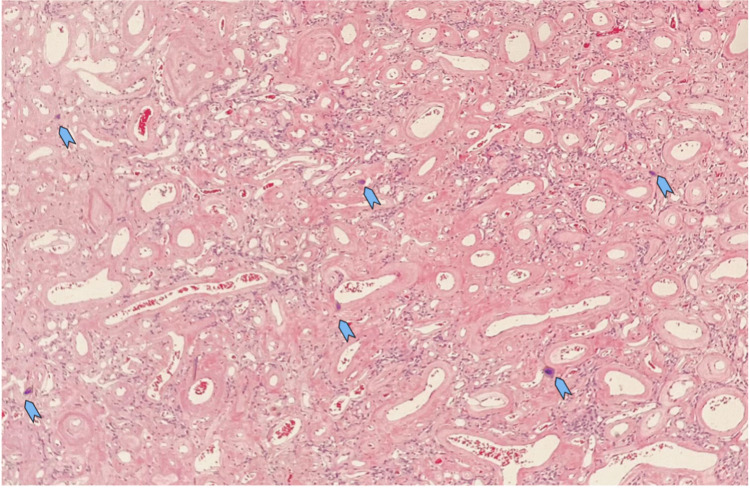
The vascular channels are variable in size with a thickened hyalinised wall. There are several foci of tiny psammoma bodies noted (arrow head)

In Figure 4, there are several foci of tiny psammoma bodies noted. Focal infiltration into dura and adjacent glial tissue is observed. On immunohistochemistry, the meningothelial cells are focally positive for the epithelial membrane antigen (EMA) and negative for S100. The vascular channels were highlighted with CD34 marker. Ki67 is very low (<25).

The patient was on ventilatory support postoperatively, and passed away 10 days later due to a cerebrovascular accident and postoperative complications.

## Discussion

OGM is a slow-growing tumour, arising from the anterior cranial base, often found at the cribriform plate of the ethmoid, fronto-sphenoidal suture and planum sphenoidale [5]. It may manifest as visual disturbances, headaches, personality changes and anosmia [6]. Headaches followed by personality changes are the most common presenting symptom [1]. Visual impairment occurs in 24-61% of cases of OGM [2]. Symptoms may present insidiously before presenting with clinical signs and the tumour may grow progressively large.

This case report of an OGM measuring 4.8 x 5.0 x 4.8 cm shows that a large tumour may exist preceding any neurological deficit. Vision may be affected usually after an OGM has become considerably large but may relate to its origin [7]. Due to the tumour's proximity to the optic nerve, progressive vision loss presenting over months to years is a common presentation. Ocular involvement occurs when there is axoplasmic flow disruption and optic nerve demyelination due to long-standing compression. It may also be caused by compression of the carotid vessel branches leading to reduced blood flow and optic nerve ischemia.

Optic nerve compression may lead to optic nerve head oedema and progress to optic atrophy and may present as Foster-Kennedy syndrome [7]. Due to the large size of OGM, it causes damage via downward pressure on the optic nerve and upward pressure on the optic chiasma [7].

Our patient had anosmia and during examination, he had difficulty comprehending certain instructions. Subtle behavioural changes may result in delayed ophthalmic care until advanced vision deterioration. The patient had a delay in seeking treatment due to poor social support. OGM tend to grow to a considerable size before its actual presentation due to symptoms such as anosmia and behavioural changes are commonly overlooked [8].

The mainstay of treatment is tumour resection with radiotherapy as an alternative option. The risk of surgery depends on the surgical approach. Tumour resection can be achieved via several approaches such as bi-frontal approach, unilateral approach, fronto-orbital approach, pterional approach, lateral supraorbital approach, and endoscopic endonasal trans-cribriform approach [2]. The surgical aim is tumour devascularisation, frontal lobe preservation, optic nerve protection, and tumour dissection from the anterior cerebral [9]. Predisposing factors for tumour recurrence and complications are age, tumour size, tumour texture and ethmoid bone infiltration [1,10].

The commonest post-surgery complications are anosmia and cerebrospinal fluid leakage [1]. In literature, bi-frontal approaches permit adequate exposure of large olfactory groove meningioma and of the ethmoidal region, and promote excellent cranial base closure, reducing cerebrospinal fluid leaks [1,4,5]. However, it has the highest rate of tumour recurrence [1]. The morbidity risk of OGM tumour resection is high, especially with larger tumours and the presence of perilesional oedema [6].

## Conclusions

Low vision and anosmia can be early signs of OGM. In patients with an advanced frontal lobe lesion, it may delay the patient’s help-seeking behaviour. OGM is a benign tumour with a good prognosis if detected early. Neuro-ophthalmologic findings should be assessed conscientiously to avoid a delay in diagnosis and treatment in order to save lives and yield favourable visual outcomes.
